# A small molecule screen to identify regulators of *let-7* targets

**DOI:** 10.1038/s41598-017-16258-9

**Published:** 2017-11-21

**Authors:** J. Cinkornpumin, M. Roos, L. Nguyen, Xiaoguang Liu, X. Gaeta, S. Lin, D. N. Chan, A. Liu, R. I. Gregory, M. Jung, J. Chute, H. Zhu, W. E. Lowry

**Affiliations:** 1 Department of Molecular Cell and Developmental Biology, UCLA, California, USA; 2 Eli and Edythe Broad Center for Regenerative Medicine, UCLA, California, USA; 3 Jonsson Comprehensive Cancer Center, UCLA, California, USA; 4 Molecular Biology Institute, UCLA, California, USA; 5 MSTP Program, David Geffen School of Medicine, UCLA, California, USA; 6 Stem Cell Program, Boston Children’s Hospital, Massachusetts, 02115 USA; 7 Department of Biological Chemistry and Molecular Pharmacology, Harvard Medical School, Boston, Massachusetts 02115 USA; 8 University of Texas-Southwest Medical Center, Texas, USA; 9 Division of Hematology and Oncology, Department of Medicine, UCLA, California, USA; 10 Department of Chemistry, UCLA, California, USA

## Abstract

The *let-7* family of miRNAs has been shown to be crucial in many aspects of biology, from the regulation of developmental timing to cancer. The available methods to regulate this family of miRNAs have so far been mostly genetic and therefore not easily performed experimentally. Here, we describe a small molecule screen designed to identify regulators of *let-7* targets in human cells. In particular, we focused our efforts on the identification of small molecules that could suppress *let-7* targets, as these could serve to potentially intercede in tumors driven by loss of *let-7* activity. After screening through roughly 36,000 compounds, we identified a class of phosphodiesterase inhibitors that suppress *let-7* targets. These compounds stimulate cAMP levels and raise mature *let-7* levels to suppress *let-7* target genes in multiple cancer cell lines such as *HMGA2* and *MYC*. As a result, these compounds also show growth inhibitory activity on cancer cells.

## Introduction

Lin28A and Lin28B are RNA-binding proteins with two types of nucleic acid interacting domains: a cold shock domain (CSD) followed by two repeats of CCHC-type zinc-binding motifs^1^. Structural analysis revealed that these domains bind to the stem loop and the GGAG domains of *let-7* precursors respectively, allowing specific interactions with various *pre-let7* members^2,3^. Spatially, it has been suggested that Lin28B is localized in the nucleus and Lin28A resides mostly in the cytoplasm^4^. Lin28B has been proposed to chaperone primary *let-7* (*pri-let-7*) in the nucleolus and away from the processing machinery, thus inhibiting its maturation. In the cytoplasm, Lin28A recruits the TUTase Zcchc11 to inhibit the maturation of precursor *let-7* (*pre-let-7*)^5^. These *let-7* family members of miRNA are known to regulate developmental timing and cell-fate decisions in less complex organisms^6,7^. *let-7* family members have identical seed sequences and divergent stem-loop regions. Their targets include many oncogenes (C-*MYC, N-MYC, RAS*, and *HMGA2*), cell-cycle regulators (*CYCLIND1, D2*), as well as other developmental regulators including *LIN28A* and *LIN28B* ^8,9^. Their mutual inhibition with Lin28 forms a powerful regulatory loop that is thought to have broad effects on developmental maturity.

Overexpression of Lin28A has been shown to delay the onset of puberty in mice as well as affecting developmental traits such as height. LIN28A has also been employed for reprogramming somatic cells to pluripotency^10^. Our own data and that of many others has shown that the LIN28/*let-7* circuit can be exploited to regulate developmental progression in various murine and human tissues^11^. On the other hand, because *let-7* activity is typically diminished in human tumors, any reagents that could block the induction of *let-7* targets would potentially be important to the treatment of cancer.

Currently, the experimental approaches employed to modulate LIN28 activity includes RNAi or overexpression; whereas *let-7* activity can be induced by transfection of *let-7* mimics or suppressed by antagomirs^12^. However these approaches can be difficult to apply especially *in vivo*. In addition to its role in development, LIN28 has also been shown to be activated in 15% of human tumors and its expression correlates with tumor progression and poor prognosis^13^. The targets of *let-7* include oncogenes as well as genes frequently found upregulated in tumors (LIN28 itself is a target of *let-7*), therefore, *let-7* may have tumor suppressive effects. Indeed, loss of function of *let-7* has been linked to cancer formation in murine models^8^. Our own data show that cells carefully titrate *let-7* activity to prevent cancer formation. It is possible that by downregulating LIN28B and/or upregulating *let-7* activity, cancer progression can be reversed. We posited that it should be possible to use small molecules to modulate levels of *let-7* targets to influence differentiation or the progression of cancer^14^. Such a therapeutic potential can be best realized by the development/discovery of bioavailable small molecules. Here we describe small molecule screening for compound that affect the expression of *let-7* targets.

## Results

### Generation of a Huh7 cell line stably expressing a *let-7* activity reporter

**Figure 1:**
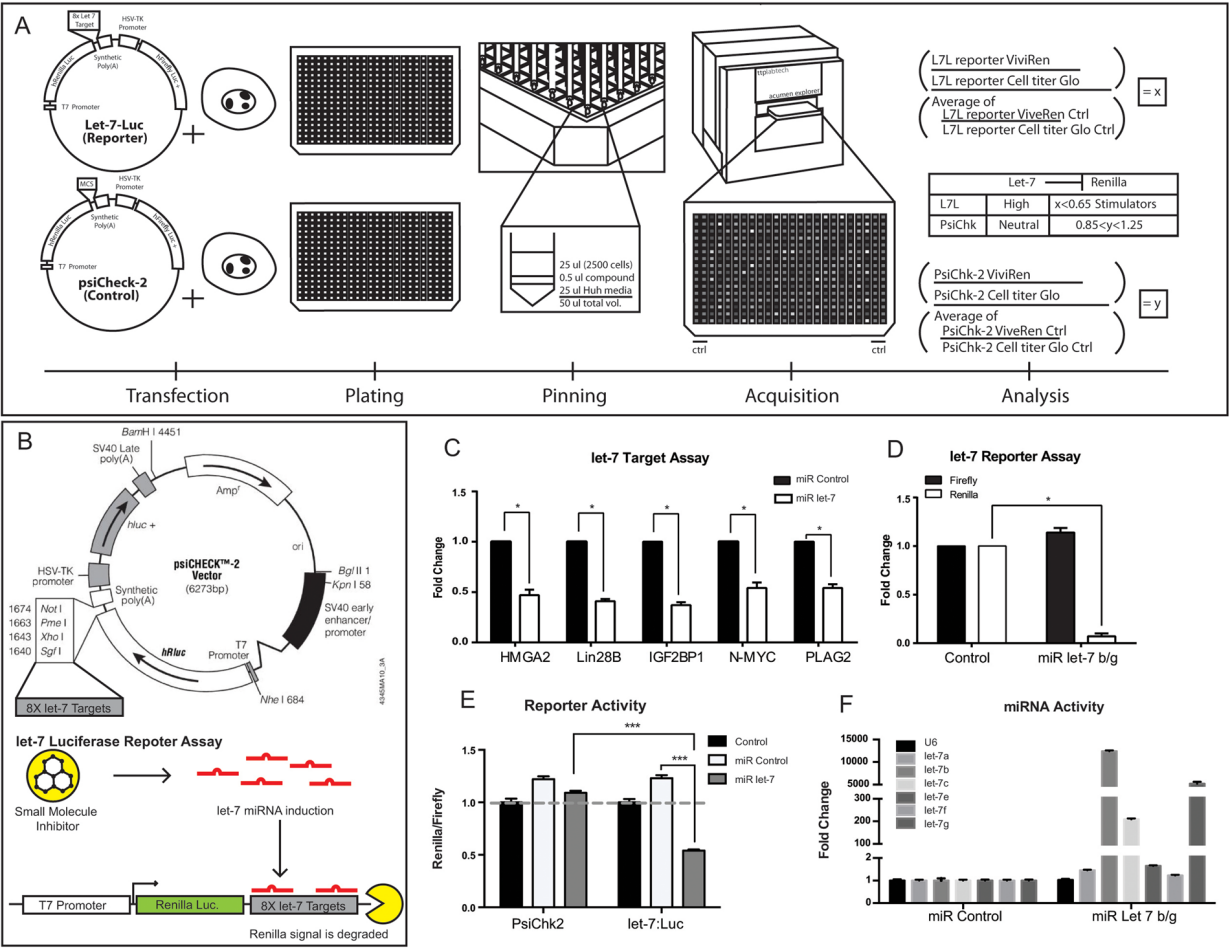
Design of screen to identify regulators of *let-7* activity. (**A**) Schematic of the *let-7* Luciferase Screen. Human liver cancer cell line (HUH) is transfected each with the *let-7*: Luciferase and PsiCheck2-control reporter plasmids. Each transfected line is plated on its own 384 plate and both pinned with its respective compound set. The treated cells are incubated at 37 C for 48 hours. ViviRen reads out the Renilla luciferase and Cell Titer Glo is a readout of cell health. (**B**) Schematic of the *let-7* Luciferase Assay. Psicheck2 plasmid was manipulated to contain the *let-7* seed sequence 8 times in tandem and linked to the renilla sequence. Therefore, when *let-7* activity is increased, the renilla luminescence will be decreased. (**C**) Transfection of *let-7* mimics silences a variety of *let-7* target genes as measured by RT-PCR. (**D**) Example of the fidelity of the *let-7* reporter construct. (**E**) Cells transfected with the *let-7* mimic showed a reduction in the readings of renilla, but the constitutive firefly luciferase was stable. (**F**) RT-PCR for mature *let-7s* in response to *let-7* transfection demonstrated the efficacy of the induction of *let-7* levels. All RT-qPCR experiments are graphed as mean +/− s.e.m. (n = 3), *p < 0.05, **p < 0.01, ***p < 0.001.

We and others have shown that *let-7* activity can be precisely assayed using a luciferase-based method (PSI-Check2 *let-7* 8X, Fig. 1A). In short, the Renilla luciferase is flanked by 8 repeats of *let-7* target sequence and therefore its mRNA will be subject to a higher rate of degradation in the presence of a higher *let-7* activity. The control Firefly luciferase was driven by a constitutive promoter (Fig. 1A). We analyzed a handful of breast cancer and hepatocarcinoma cell lines (MCF7, MCF15, Huh7 and Huh7.5.1) to assay the detectable *let-7* activity (Fig. S1A). In Human Hepatocarcinoma (Huh). We observed a high level of LIN28B expression at both the RNA and protein level (Fig. S1B and C); and as a result, a low level of *let-7* activity, as shown by *let-7*-luc luciferase assay (Figure S1A). In addition, we found that the Huh cell line expressed a number of *let-7* targets that could be tightly regulated by changes in *let-7* levels (Fig. 1C).

To facilitate reproducible results in both screening and validation assays, we created a cell line with stable integration of the *let-7* reporter construct. We cloned a Neomycin resistance cassette into the PSI-Check2 *let-7*-luciferase, and then stably introduced the reporter plasmid into the Huh7.5.1 cell line and selected with G418 for 3 weeks (Fig. 1B). The stable cell line was subjected to dual-glo luciferase assay, where it displayed a stable luciferase unit per cell in both Firefly and Renilla (Fig. 1D). To demonstrate the dynamic range of detection in *let-7* activity, we transfected this Huh7.5.1 *let-7* luciferase reporter line (Huh7.5.1 L7L) with siRNA against LIN28B (Fig. S1F), as well as *let-7* mimics (Fig. 1D). siRNA effectively reduced LIN28B expression by at least 90% (Figure S1B and C). In response to the downregulation of LIN28B, mature microRNA levels rose about 2 to 3 fold for all *let-7* family members (Figure S1D). As a result, the *let-7* activity was reduced by 25–50%, as assayed by dual-glo luciferase (Figure S1F). In addition, we used transfection of mimics of let-7s to determine how sensitive the reporter was to changes in let-7 levels (Fig. 1E and F). This demonstrated that strong induction of let-7 levels by direct transfection was able to effectively silence the reporter (Fig. 1E).

### High Throughput Screening of Small Molecules

The initial screens with the *let-7* reporter stably introduced into Huh cells generated significant numbers of false positives in both directions. As expected, many of the false positive appeared to target luciferase enzymes, and not *let-7* activity. As an alternative method designed to minimize the identification of molecules that target luciferase, we transiently transfected replicate wells with a PSI-Check2 plasmid that either contained the *let-7* seed sequence or a clean version that should not be regulated by *let-7*. We then quantified the signal change in the screen as a function of the effect on the luciferase without *let-7* sites (Fig. S1E and H), and as a function of internal controls on each reporter consisting of alternate luciferase gene (firefly) driven by a constitutive promoter. As a result, we were able to screen for molecules that affected *let-7* activity directly, after controlling for both luciferase and transfection efficiency (Fig. 1A). We also validated the assay protocol based on its performance for high throughput screening (HTS) suitability^15^. For the optimized HTS reporter screen we derived a Z’-factor of 0.65 which is indicative of a reliable assay activity^15^. We screened through roughly 36,480 small molecules as described in Materials and Methods, and uncovered at least 60 potential candidate molecules.

### Screening using expression of let-7 target genes

**Figure 2:**
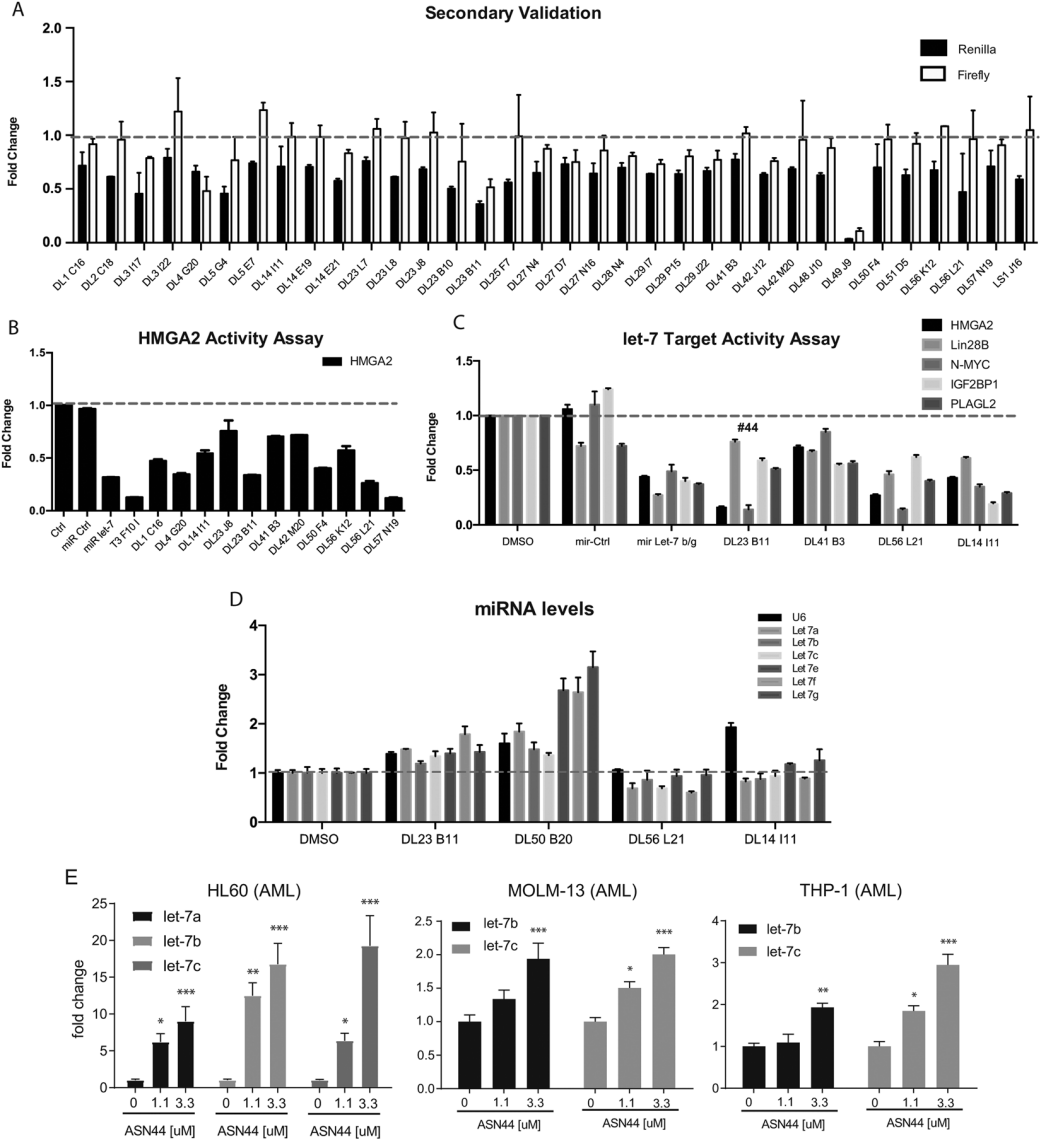
A secondary screen to Identify compounds that suppresses *let-7* targets. (**A**) A secondary screen using both constitutive and *let-7* sensitive reporter to identify high confidence hits. (**B**) tertiary screening of compounds for effect on expression of HMGA2, an established *let-7* target gene by RT-PCR. (**C**) RT-PCR for additional *let-7* target genes after treatment with compounds that came out of tertiary screen. (**D**) RT-PCR for small RNAs to determine whether any compounds regulate *let-7* levels. (**E**) RT-PCR for detectable mature *let-7* family members in AML cell lines in response to treatment with two doses of #44. All RT-qPCR experiments are graphed as mean +/− s.e.m. (n = 3), *p < 0.05, **p < 0.01, ***p < 0.001.

We proceeded to validate the top potential *let-7* stimulators and potential *let-7* inhibitors. Using small aliquots provided by the screening facility, we carried out primary validation on Huh7.5.1 L7L in a 48-well format using a dual-glo luciferase assay on the first 29 hits. After eliminating false positive hits, several potential *let-7* stimulators and inhibitors remained (Fig. 2A). While this approach was useful to narrow the list of candidates, we found in subsequent experiments that many of the candidates passing this secondary screen either had small or highly variable activities on *let-7* activity when judged by relative amounts of *let-7* target genes (data not shown). In essence, the high sensitivity of the luciferase method was unable to distinguish the candidates with physiologically significant activity.

To identify candidate regulators of *let-7* activity from the screen more directly, we performed a tertiary screen that measured levels of the *let-7* target *HMGA2*. We chose this gene because it is expressed in several different isoforms, only one of which has more than one *let-7* target site in its 3′ UTR. By quantifying the relative expression of the *HMGA2* isoform with many *let-7* sites versus all *HMGA2* isoforms, we could identify specific activation of *let-7* activity without the use of an exogenous reporter. We assayed 60 candidates from the original screen (Fig. 2). With this screening approach, we confirmed several of the candidates that were validated by luciferase in Fig. 2A, and also identified additional compounds able to directly affect expression levels of the long form of *HMGA2* (Fig. 2B). This led to the identification of a compound we labeled #44. The structure and purity of compound #44 were confirmed by 1 H and 13 C NMR and TLC. The NMR data and spectra were included in Fig. S2.

We tested by RT-PCR whether *IMP3 (IGF2BP3), PLAG2, L*I*N28B* or *MYC* were affected by treatment of HUH7 cells with #44 (Fig. 2C). In Huh7 cells, #44 appeared to suppress the expression of *HMGA2, N-MYC*, and *IMP2*, while *LIN28B* and *PLAG2* did not seem to change significantly (Fig. 2C). The fact that 3 out of 5 *let-7* targets were suppressed by #44 could suggest that *let-7* activity is induced in these cells, and *let-7* levels are altered depending on their endogenous expression levels. As *let-7* miRNAs are highly expressed in Huh7 cells, endogenous changes of mature let-7miRNA levels are difficult to detect. Perhaps consistent with this notion, treatment of cells with #44 did not have a strong impact on the level of mature *let-7*s (Fig. 2D).

To determine the general applicability of #44 to influence let-7 target expression, we measured the effect of this compound on various Acute Myeloid Leukemia (AML) cell lines each with well-characterized expression levels of *let-7s* and *LIN28*. Most AML cell lines do not express high levels of *let-7* miRNA levels. Perhaps as a consequence, treatment of AML cells significantly upregulated mature *let-7* levels in MOLM-13, THP-1 and HL60 cell lines (Fig. 2E).

**Figure 3:**
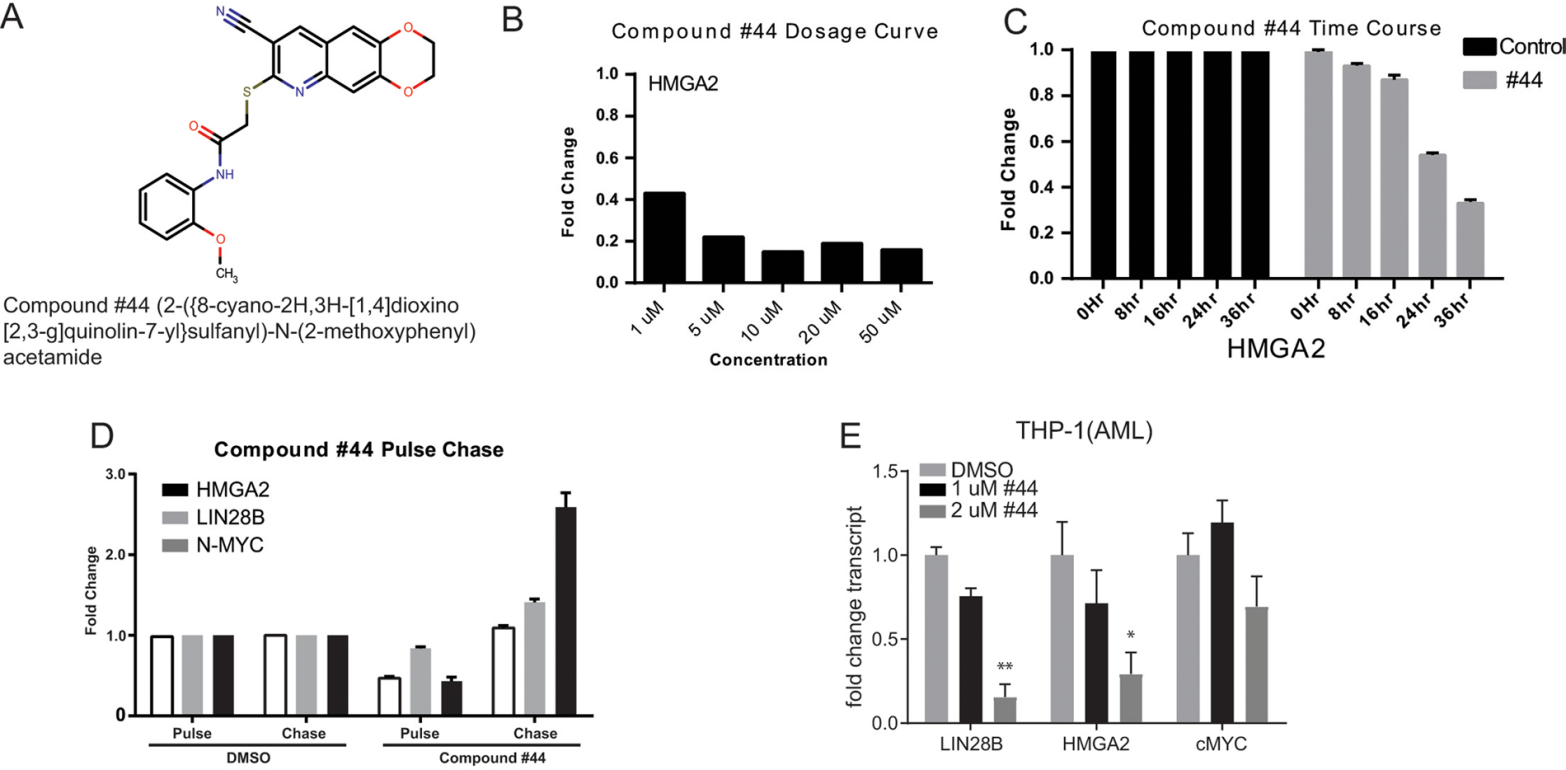
Identification of compound that reliably suppresses expression of *HMGA2*. (**A**) The structure and chemical name of #44. (**B**) A dose curve of treatment with #44, as measured by the expression level of *HMGA2*. (**C**) Treating Huh7 cells with #44 at 1uM for various times showed specific activity at just 8 hours. (**D**) Treatment with #44 for two days showed specific suppression of *HMGA2*-long (PULSE), whereas two days after removal of these compounds, levels of *HMGA2* returned to baseline (CHASE), indicating the effect of the compound is reversible. (**E**) RT-PCR for let-7 target genes in response to #44. All RT-qPCR experiments are graphed as mean +/− s.e.m. (n = 3), *p < 0.05, **p < 0.01, ***p < 0.001.

Focusing on the most reliable suppressor of *HMGA2*, #44 (Fig. 3A), we performed dose curve, time course, and pulse-chase experiments (Fig. 3B-E). Varying concentrations of these compounds were applied to Huh7 cells and assayed by RT-PCR for the *let-7* sensitive version of *HMGA2*. These dose curve experiments showed that #44 was effective at 1uM, and maximally effective at 5uM (Fig. 3B). To determine the time course for activity of #44, cells were treated for various times. RT-PCR for *HMGA2* showed that #44 could suppress expression of this *let-7* target gene in as few as 8 hours (Fig. 3C). Finally, we performed a pulse-chase of treatment with #44 to determine if the effect on *let-7* targets was permanent or instigated a feed forward program of suppression of *let-7* targets. In fact, treating with #44 for 2 days followed by treatment withdrawal for 2 days completely reversed the effect of this compound on various *let-7* target genes (Fig. 3D), suggesting that this compound transiently regulated expression of *let-7* targets. Treating a AML cell line with #44 also showed a dose-responsive effect on those let-7 targets that are expressed (Fig. 3E).

### Identification of a potential target pathway for #44

**Figure 4:**
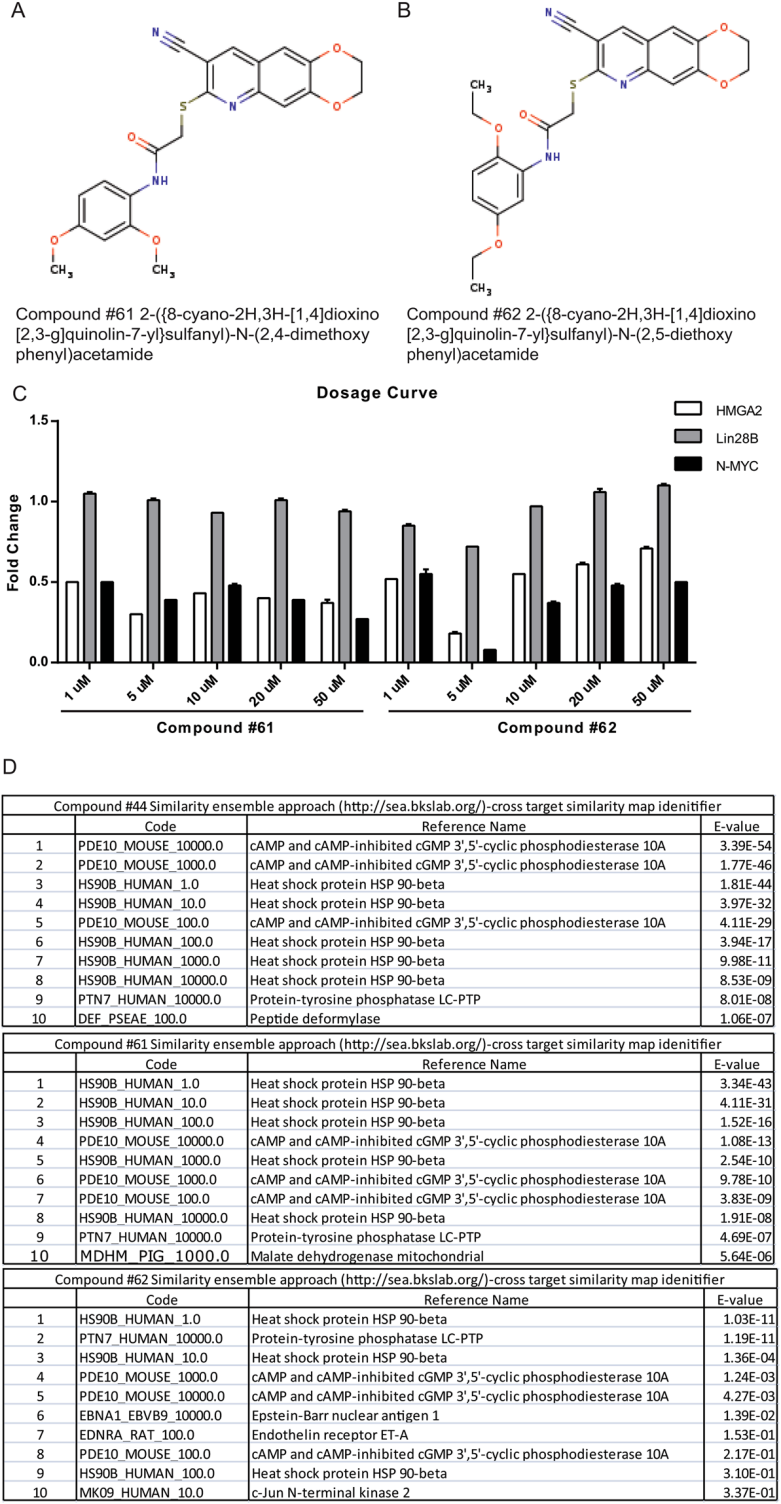
Identification of analogues of #44 (**A**) and (**B**). Two chemical analogues of #44 from the same library show similar structure. (**C**) #61 qnd #62 show similar effect on expression of let-7 targets such as *HMGA2, NMYC* and *LIN28B*. (**D**) SEA analysis predicts the top targets of these compounds as PDE inhibitors.

Based on the structure of #44, we identified several very similar molecules that would be predicted to have the same effect (designated #61 and #62) (Fig. 4A and B). Treatment of Huh cells with these compounds also suppressed levels of *HMGA2* and *NMYC* (Fig. 4C).

**Figure 5:**
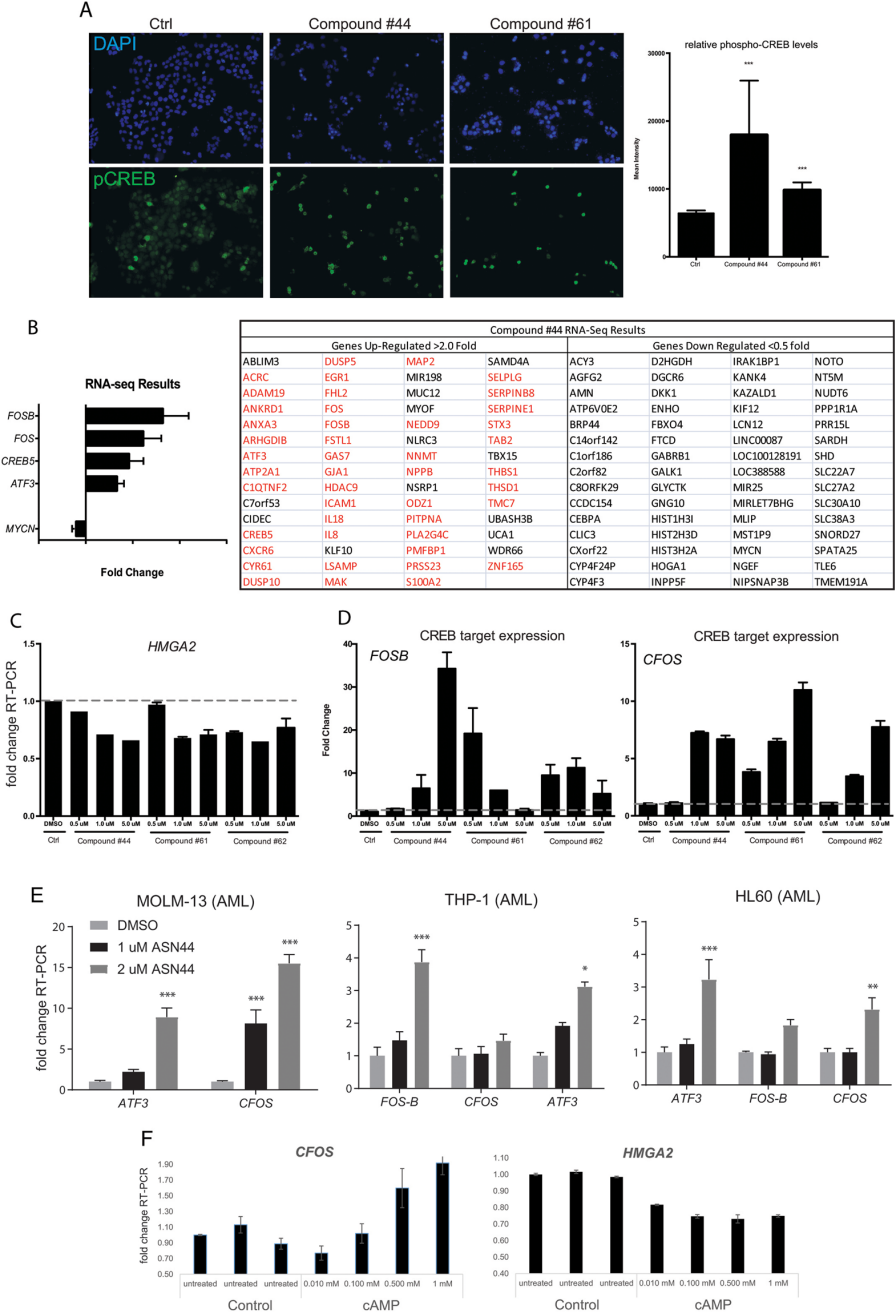
#44, 61 and 62 are PDE inhibitors that activate CREB. (**A**) Huh cells were treated with #44 or #61 for 48 hours and then immunostained with an antibody that recognizes the phosphorylated version of CREB, which is typically translocated to the nucleus when active. Quantification shown on the right. (**B)** RNA-sequencing of Huh cells treated with #44 in triplicate showed strong induction of CREB target genes (*FOSB, FOS, CREB5, and ATF3*). A list of genes up and downregulated by at least 2 fold in response to treatment with #44. Those highlighted in red were previously shown to be direct targets of CREB in another study. (**C**) Dose response of #44, 61, 62 for an effect on *let-7* target gene, *HMGA2*. (**D**) RT-PCR for direct Creb targets *FOSB* and *CFOS* shows that #44, 61, and 62 all stimulate CREB target gene expression. (**E**) RT-PCR for CREB target genes in AML cell lines in response to treatment with #44. (**F**) Directly treating HUH cells with cAMP stimulates this pathway as measured by induction of CREB target (*CFOS*), and *HMGA2* was downregulated as a result as measured by RT-PCR. All RT-qPCR experiments are graphed as mean +/− s.e.m. (n = 3), *p < 0.05, **p < 0.01, ***p < 0.001.

We looked for targets of #44, 61, and 62 by inputting their structures into SEA viewer, structure-target prediction software^16^. This led to identification of Phosphodiesterase 10 A as a potential target of #44, 61 and 62. The role of phosphodiesterase is to regulate levels of cyclic-AMP (cAMP) (Fig. 4D). Therefore, if #44 inhibits PDE10, one would expect an increase in cAMP levels leading to cAMP Responsive Element Binding protein (CREB) activation. Huh cells were treated with #44 and #61 and then stained with an antibody that recognizes phosphorylated CREB, consistent with activation of cAMP signaling. Both #44 and #61 induced levels of nuclear phosopho-CREB, which is an established method to detect active cAMP signaling (Fig. 5A). To measure complete degree to which #44 could regulate gene expression in Huh cells, we carried out RNA-seq to identify which genes are changed in response to treatment with these compounds and whether *let-7* targets are enriched amongst these gene expression changes (Fig. 5B). A wide variety of genes appeared to be both induced and suppressed. At the top of the list were genes related to CREB signaling, particularly induction of *ATF3, C-FOS and FOSB*, suggesting that #44 activates CREB (Fig. 5B). We also performed dose-response assays on cells treated with #44, 61 and 62. These compounds again appeared to silence HMGA2 in a dose dependent manner (Fig. 5C), but also these compounds induced typical CREB target genes such as FOSB and CFOS in Huh7 cells and *ATF3, FOSB and CFOS* in HL60, MOLM13 and THP-1 cells (AML lines) (Fig. 5D and E). Finally, treatment of HUH cells with cAMP itself also led to a downregulation of HMGA2 (Fig. 5F), further suggesting that at least some let-7 target genes are regulated by cAMP signaling.

**Figure 6:**
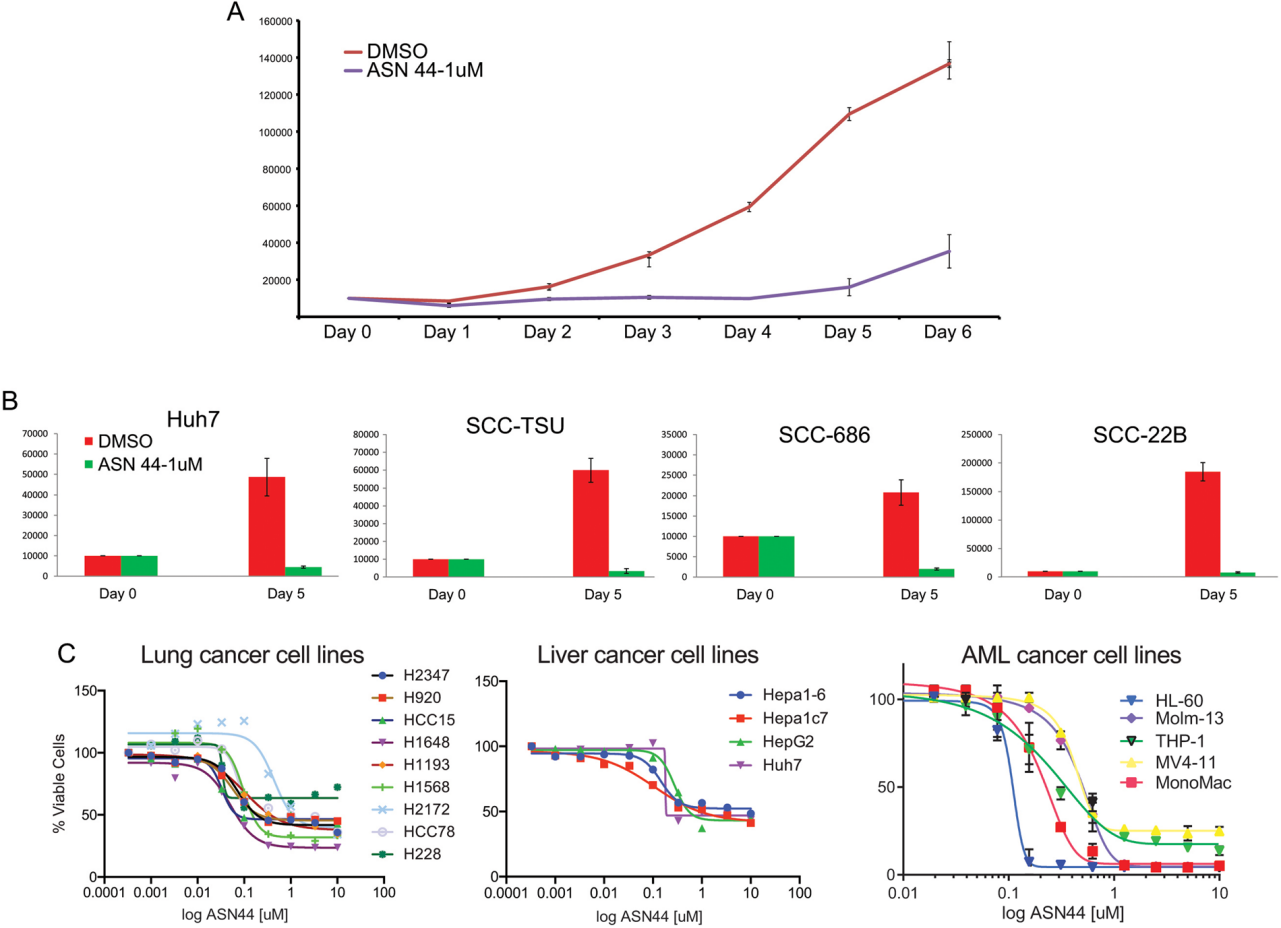
Extended treatment of cancer cells with *let-7* inducing compounds blocks their growth. (**A)** Equal numbers of Huh were plated across replicate wells and treated with the indicated compounds. Each day, several wells of each treatment condition were counted. Low dose of #44 slowed the growth of this cancer cell line. (**B**) Treatment of Huh and three human squamous cell carcinoma cell lines (TSU, 22B, and 686) also showed a strong effect of #44 on cell growth at 5 days of treatment. (**C**) Various lung, liver, and AML cancer cell lines were plated with escalating doses of #44, and cell viability was assayed by ATP-luciferase at 48 hours. All growth proliferation assays are graphed as mean +/− SD (n = 3), *p < 0.05, **p < 0.01, ***p < 0.001.

Because the compounds showed the ability to suppress expression of proliferation-associated genes such as *HMGA2* and *NMYC*, we posited that #44 might affect the growth rate of cancer cell lines. To determine the physiological effect of *let-7* stimulation by the candidate hits, Huh and other cancer cell types were treated over longer time courses to allow for measurements of growth kinetics. #44 appeared to dramatically slow the growth of Huh cells at 1uM, the same dose used to effectively suppress *let-7* targets (Fig. 6A). We then extended these analyses to squamous cell carcinoma lines and observed the same effect of #44 (Fig. 6B). Additionally, with a distinct cell growth assay, compound #44 showed significant toxicity towards several lung, liver, and AML cancer cells lines with an IC50 of just 0.1uM (Fig. 6C). These data are actually consistent with previous studies showing that PDE inhibition can be an effective mediator of growth rate in cancer cell lines^17,18^.

## Discussion

We have generated a cell-based model suitable for high throughput small molecule screening for *let-7* activity modulators in 8 small molecule libraries. We generated a stable *let-7*-luciferase reporter line (Huh7.5.1 L7L), which expresses far less luciferase mRNAs (and proteins) than transiently transfected cell lines. Since this luciferase system reports for *let-7* mediated degradation of the Renilla luciferase mRNA, it allowed a higher sensitivity for any reagents that can modestly change the *let-7* activity. We also encountered the problem of non-specific effects of small molecules on luciferase readings during the high through-put screening, primary and secondary luciferase validations. This was addressed by comparing the psiCHECK2- *let-7* 8 × luciferase reporter and the psiCHECK2 control luciferase reporter during the screening process to weed out false positive hits and prevent the loss of false negatives. Alternatively, a fluorescence-based reporter on *let-7* activity should also be considered in future screening efforts to reduce false positives due to inhibition or stimulation of the luciferase enzyme itself.

The PDE inhibitors identified in this screen showed an effect on the growth of cancer cell lines. Cancer cells have been show to exhibit reduced malignancy and motility when LIN28 is suppressed and *let-7* activity is elevated^13^. Furthermore, *let-7* activity is tightly controlled to ensure appropriate regulation of their target genes, and misregulation of *let-7* is strongly associated with inappropriate growth of the liver^19^. Moreover, the approach outlined here can be exploited to find regulators of other miRNA families by simply changing the seed sequences in the 3′ UTR of luciferase.

## Materials and Methods

The High Throughput Screen (HTS) measures renilla luciferase expression as a function of *let-7* activity in *let-7* luc transfected Huh7 cells. Compounds from diverse chemical libraries (Biomol, Prestwick, Emerald, Microsource, NIH Clinical Collection, UCLA Lead-Like Compound Set (LS), and UCLA Diverse Library (DL)) provided by the staff of UCLA’s Molecular Screening Shared Resource (MSSR) were assayed (full details available at http://www.mssr.ucla.edu/libraries.html). Compounds were added to individual wells of either Psi-check2 transfected Huh7 cells or Psi- *let-7* transfected Huh7 cells. The MSSR plated compounds in a 384-well Matrix tissue culture treated plates (Thermo Scientific, 4334-11) with a Biomek FX liquid handler. Huh 7 transiently expressing *let-7* luc and Psi-Check2 line cells in were grown in standard Huh media including: DMEM High Glucose (Invitrogen), 10% FBS (HyClone), 1% HEPES Buffer (Invitrogen), 1% NEAA (Invitrogen), 1% penicillin/streptomycin, 5ml L-Glutamine. Cells were dispensed into wells containing the compounds using a 384-well Multidrop (Thermo Lab Systems) to a concentration of 2000cells/well and a final compound concentration of 10 µM. After 48 hours incubation, Renilla Luciferase expression was evaluated through addition of the live-cell luciferase reagent, ViviRen (Promega, 326846) at a concentration of 60uM per well by 38-well Multidrop. Luminescence was assayed using the luminometer function of an Acumen Explorer (TTP Labtech). Cell concentration per well was similarly measured using Cell Titer Glo (Promega, G755A) in which we use to normalize the levels of renilla luciferase readings. Data from the screen was uploaded to the Collaborative Drug Discovery platform www.collaborativedrug.com, where hit identity was established. Compounds were considered hits only after signals were normalized for *let-7* luc relative to the normalized Psi-Check2 reading. In addition, luciferase signals were normalized to levels of cell titer GLO (Promega) in each well to rule out compounds with high toxicity.

### Cell Culture

Huh7, Huh7.5.1, Huh7.5.1 L7L were cultured as previously described^20^. Briefly, these cells are cultured in DMEM Hi-Glu (Life Technologies) supplemented with 10% inactivated FBS (Hyclone), 1X P/S-Glu, and 1X HEPES (Life Technologies). HL-60, MOLM-13 and THP-1 cell lines were obtained from ATCC and cultured at low passage numbers in Roswell Park Memorial Institute (RPMI) 1640 media supplemented with HEPES, L-Glutamine, 10% fetal bovine serum and 1% Penicillin/streptomycin (Life Technologies).

### Reporter assay

Cells transfected with the psiCHECK2- *let-7* 8 × luciferase reporter (Addgene #20932) or psiCHECK2 control reporter (Promega) were dissociated 72 h post-transfection, treatment, and then subjected to dualglo luciferase assay as described in the manufacturer’s protocol (Promega). The Renilla luciferase gene was driven by T7 promoter and contained eight *let-7* targeting sequences in the 3′ UTR, and Firefly luciferase driven by a constitutive promoter as a transfection control. Luciferase assays were carried out in a GloMax 96 Microplate Luminometer (Promega). The assay quality was estimated by the Z’factor formulae Z’ = 1 − [(3 × (SDpositive control + SDnegative control)/(averagepositive control − averagenegative control)]. Positive controls were luciferase readouts of let-7 mimics, negative controls were luciferase signals from control treated cells.

### Selectable let-7 activity reporter

The PBabe Neo plasmid (Addgene #1767) was linearized with the restriction enzyme BamHI (New England BioLabs). The Amplicin-resistance cassette in psiCHECK2- *let-7* 8X was digested with BamHI and BglII (New England Biolabs) and the 5000 bp fragment containing the luciferase reporters (but no Amp^R^) was ligated with the linearized Pbabe Neo. The resulting plasmid was ligated, selected with Ampicilin, and sequenced.

### Stable reporter cell line

Huh7.5.1 was transfected with the selectable *let-7* activity reporter using Lipofectamine 2000 (Life Technologies) according to manufacturer’s protocol. The transfected cells were selected with G418 (Life Technologies) at 500–1000ug/mL for 3 weeks. The stable reporter cell line was maintained with 600–800ug/mL of G418.

### siRNA and let-7 mimic transfection

Huh7.5.1 *let-7* -luciferase line was transfected with Lipofectamine RNAiMax (Life Technologies) according to manufacturer’s protocol. siRNA against Lin28B and *let-7* mimics were purchased from Dharmacon.

### Primary Hit Validation

Compounds found in the HTS to significantly stimulate or inhibit Renilla luciferase expression, suggesting *let-7* activity regulation, were procured from the MSSR and plated at 10uM on Huh7.5.1 reporter line cells in 48-well. After 48 hour incubation, *let-7*-regulated Renilla luciferase and constitutively expressed Firefly luciferase were measured using Promega’s Dual-Luciferase Reporter Assay System and a GloMax 96 Microplate Luminometer (Promega). Validated hits were purchased from vendors [Sigma-Aldrich (http://www.sigmaaldrich.com/chemistry.html), Asinex (http://www.asinex.com/), and Molport (http://www.molport.com/buy-chemicals/index)] for further testing.

### NMR purity confirmation

Thin-layer chromatography (TLC) was performed using Silicycle silica gel 60 F254 precoated plates (0.25 mm) and visualized by UV fluorescence quenching or staining with *p*-anisaldehyde solutions. ^1^H and ^13^C NMR spectra were recorded on a NMR spctrometer-AV500 (at 500 and 126 MHz, respectively) and were reported relative to Me_4_Si (δ 0.0). Data for ^1^H NMR spectra were reported as follows: chemical shift (δ ppm) (multiplicity, coupling constant (Hz), integration). Multiplicities are reported as follows: s = singlet, d = doublet, t = triplet, q = quartet, sept = septet, m = multiplet, comp. m = complex multiplet, app. = apparent, br s = broad singlet. Data for ^13^C NMR spectra are reported in terms of chemical shift relative to Me_4_Si (δ 0.0).

## Electronic supplementary material

Supplemental Figures
