# Supplementary material for: A small molecule screen to identify regulators of let-7 targets
Source: Sci Rep. 2017 Nov 21;7:15973. doi: 10.1038/s41598-017-16258-9 (PMC5698460; doi:10.1038/s41598-017-16258-9)
Supplement: Supplementary file 1 — Supplemental Figures [file 41598_2017_16258_MOESM1_ESM.pdf]

A small molecule screen to identify regulators of *let-7* targets

Cinkornpumin J[2], Roos M[3,9], Nguyen L[8], Liu Xiaoguang[10], Gaeta X[2,5], Lin S[6,7] Chan DN[1,2], Liu A[1,2], Gregory RI[6,7], Jung M[10], Chute[3,9] J, Zhu H[8] and Lowry WE[1-4]

Supplemental Legends

Figure S1. A screening platform to identify regulators of *let-7* activity

A, Human breast cancer cell lines MCF7, MCF15 and human hepatocarcinoma cell lines Huh7, Huh7.5.1 were transfected with either PSI-Check2 or PSI-CHeck2 *let7*-8X. Dual-glo luciferase assay was performed 48hrs after transfection in triplicates. **B,** Huh cells were transfected with either non-targeting siRNA (siNT) or siRNA against Lin28B. Lysis was collected 48hrs after transfection, mRNA extraction and reverse-transcription PCR was carried out to assay the relative amount of Lin28B mRNA. **C,** Huh cells were transfected with either non-targeting siRNA (siNT) or siRNA against Lin28B. Transfected cells were re-plated on coverslips and fixed 72hrs after transfection. Immunofluorescence staining of Lin28B (red) and DNA (blue) was carried out to assay the amount of Lin28B protein. **D,** Huh7.5.1 L7L was transfected with either non-targeting siRNA (siNT) or siRNA against Lin28B. Lysis was collected 48hrs after transfection, total RNA extraction and reverse-transcription PCR was carried out to assay the relative amount of mature *let-7* miRNA. U6b and mir-15a serve as internal controls. **E, Left,** Measurement of Raw Firefly Luciferase Unit in untransfected Huh7.5.1 and Huh7.5.1 *let-7* luciferase reporter line at the two plating concentrations. **E, Middle**, Measurement of Raw Renilla Luciferase Unit in untransfected Huh7.5.1 and Huh7.5.1 *let-7* luciferase reporter line at the two plating concentrations. **E, Right,** Normalized Renilla/Firefly value in untransfected Huh7.5.1 and Huh7.5.1 *let-7* luciferase reporter line at the two plating concentration. **F,** Huh7.5.1 L7L was transfected with either non-targeting siRNA (siNT) or siRNA against Lin28B. Untransfected and transfected cells were re-plated in 20 replicates and assayed 72hrs after transfection. Viviren life cell substrate was used to assay *let7*

activity and was normalized to Cell Titer Glo to control for changes in cell number or viability. **G,** oligos that mimic mature *let-7* were transfected into Huh7.5.1 L7L, and relative *let-7* activity was measured. As expected, the reporter faithfully read out induction of *let-7* by silencing the reporter. **H**, Reporter assays demonstrate the specificity of *let-7* mimic and antagomirs in Hela cells, which do not express LIN28A or LIN28B. Reporter assays were performed with either the *let-7* sensistive dual luciferase reporter (left), or constitutive (non-*let-7* sensitive) reporters (middle). On the right is displayed the activity of the *let-7* sensitive reporter divided by that of the constitutive reporter. **I**, An accounting of screen with both the constitutive and regulatable reporters to identify small molecule regulators.

Figure S2. NMR validation of structure and purity of Compound #44.

A, traces from both 1H- and 13C-NMR to identify and quantify the purity of compound #44. B, the predicted structure of compound #44 as described in the screen and presented by the vendor (top), and a listing of masses identified by NMR (bottom).

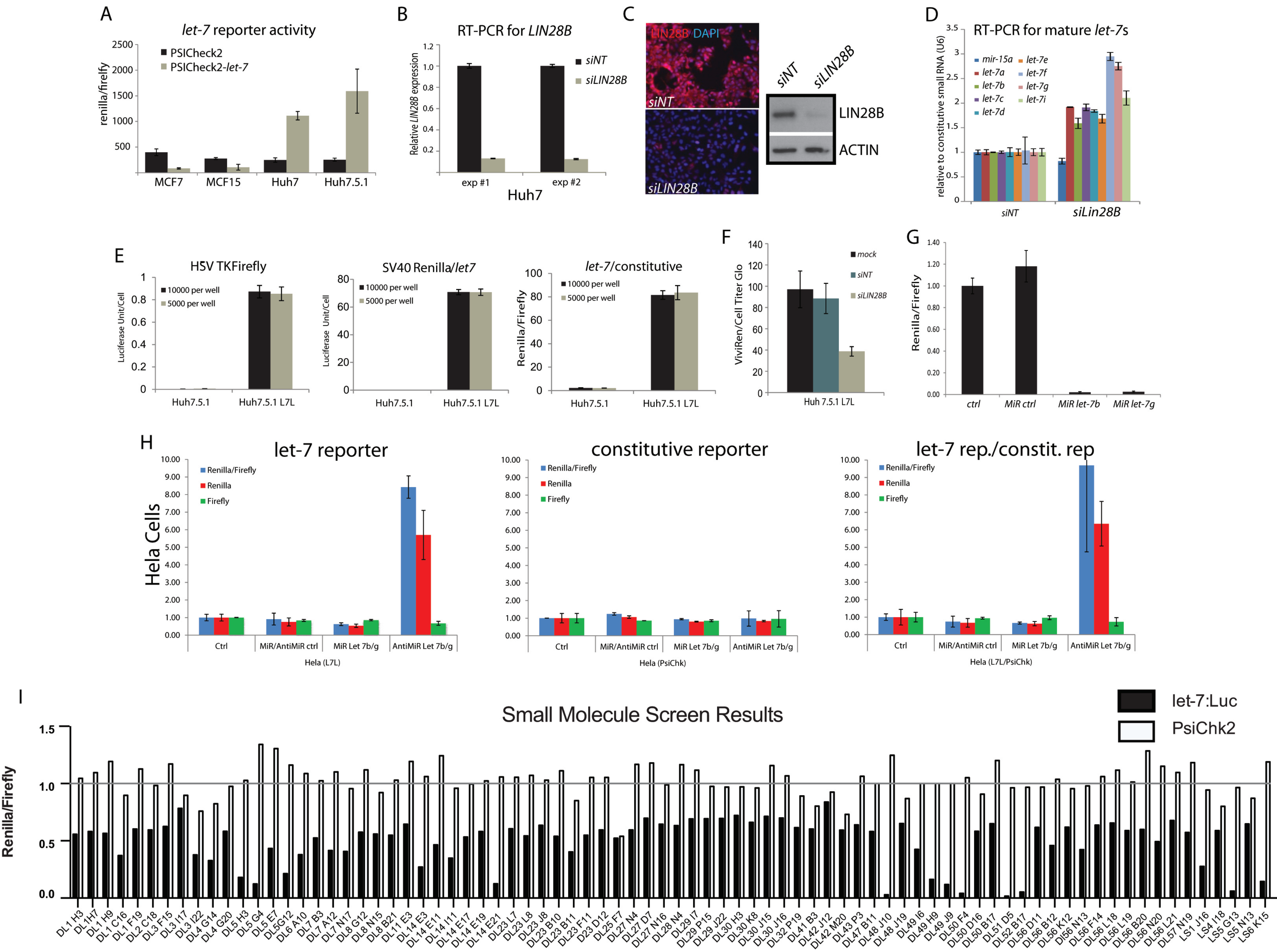
A
let-7 reporter activity
renilla/firefly
PSICheck2
PSICheck2-let-7
MCF7
MCF15
Huh7
Huh7.5.1
B
RT-PCR for LIN28B
Relative LIN28B expression
siNT
siLIN28B
exp #1
exp #2
Huh7
C
LIN28B DAPI
siNT
siLIN28B
siNT
siLIN28B
LIN28B
ACTIN
D
RT-PCR for mature let-7s
relative to constitutive small RNA (U6)
mir-15a
let-7a
let-7b
let-7c
let-7d
let-7e
let-7f
let-7g
let-7i
siNT
siLin28B
E
HSV TKFirefly
Luciferase Unit/Cell
10000 per well
5000 per well
Huh7.5.1
Huh7.5.1 L7L
SV40 Renilla/let7
Luciferase Unit/Cell
let-7/constitutive
Renilla/Firefly
F
ViviRen/Cell Titer Glo
mock
siNT
siLIN28B
Huh 7.5.1 L7L
G
Renilla/Firefly
ctrl
MiR ctrl
MiR let-7b
MiR let-7g
H
Hela Cells
let-7 reporter
constitutive reporter
let-7 rep./constit. rep
Renilla/Firefly
Renilla
Firefly
Ctrl
MiR/AntiMiR ctrl
MiR Let 7b/g
AntiMiR Let 7b/g
Hela (L7L)
Hela (PsiChk)
Hela (L7L/PsiChk)
I
Small Molecule Screen Results
let-7:Luc
PsiChk2
Renilla/Firefly

A

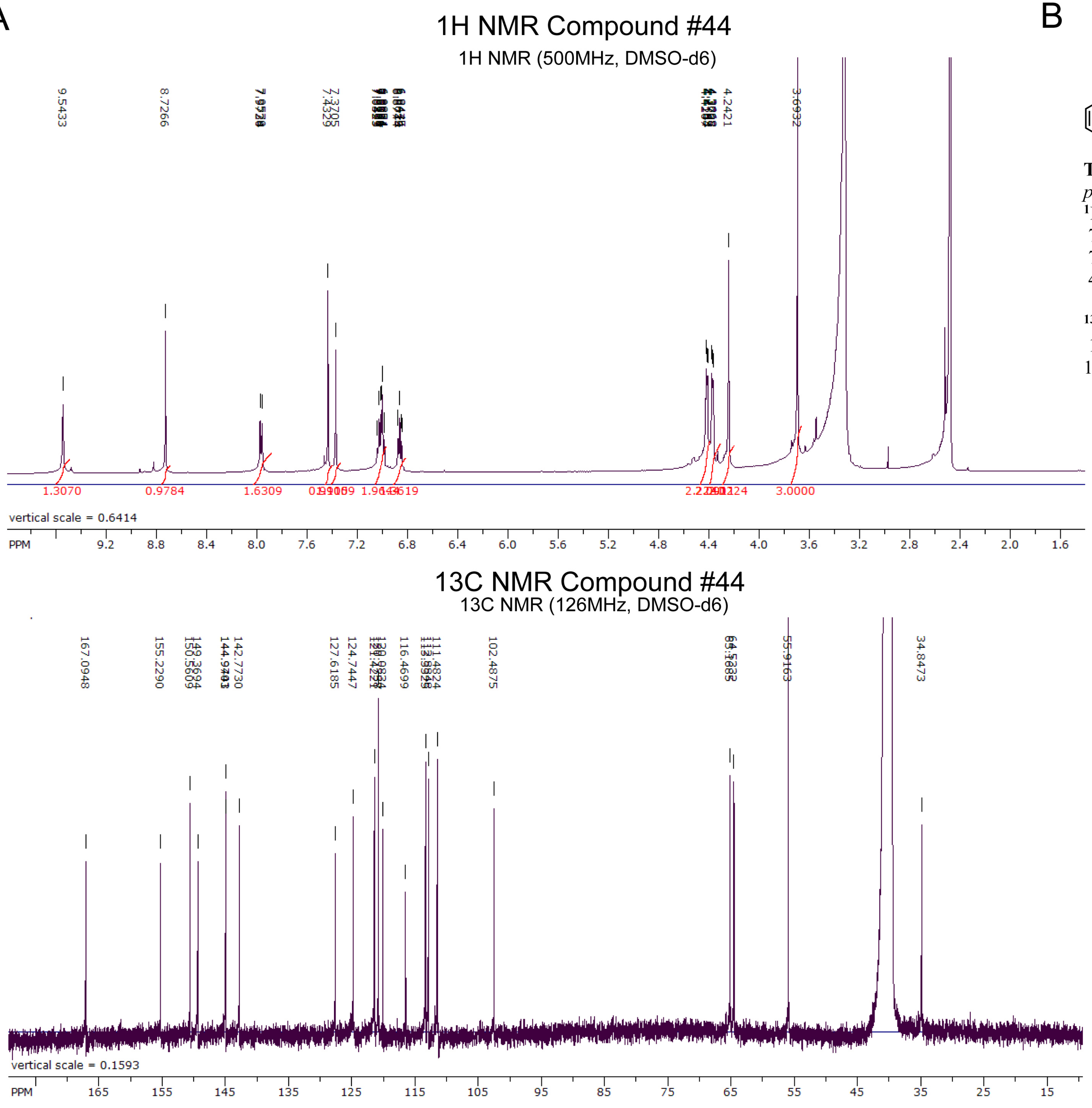

B

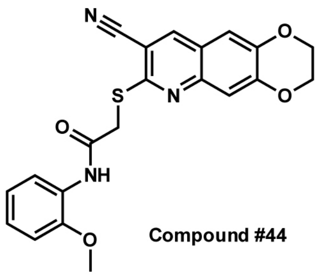

Compound #44

TLC (SiO_2) R_f = 0.42 in 1:1 hexanes/EtOAc, *p*-anisaldehyde stain and UV

^{1}H NMR (500 MHz, DMSO-d_6) δ 9.55 (s, 1H), 8.73 (s, 1H), 7.96 (d, *J* = 8.0 Hz, 1H), 7.43 (s, 1H), 7.37 (s, 1H), 7.01 (m, 2H), 6.86 (app. t, *J* = 8.0 Hz, 1H), 4.42 (m, 2H), 4.37 (m, 2H), 4.24 (s, 2H), 3.69 (s, 3H)

^{13}C NMR (126 MHz, DMSO-d_6) δ 167.1, 155.2, 150.6, 149.4, 145.0, 144.9, 142.8, 127.6, 124.6, 121.4, 120.8, 120.1, 116.5, 113.3, 112.9, 111.5, 102.5, 65.1, 64.6, 55.9, 34.9
